# Let the Body’n’Brain Games Begin: Toward Innovative Training Approaches in eSports Athletes

**DOI:** 10.3389/fpsyg.2020.00138

**Published:** 2020-02-19

**Authors:** Anna Lisa Martin-Niedecken, Alexandra Schättin

**Affiliations:** ^1^Department of Design, Subject Area in Game Design, Zurich University of the Arts, Zurich, Switzerland; ^2^Department of Health Sciences and Technology, Institute of Human Movement Sciences and Sport, ETH Zurich, Zurich, Switzerland

**Keywords:** eSports, exergaming, effectiveness, attractiveness, cognition, physical activity, performance, health

## Abstract

The phenomenon of eSports is omnipresent today. International championships and their competitive athletes thrill millions of spectators who watch as eSports athletes and their teams try to improve and outperform each other. In order to achieve the necessary cognitive and physical top form and to counteract general health problems caused by several hours of training in front of the PC or console, eSports athletes need optimal cognitive, physical and mental training. However, a gap exists in eSports specific health management, including prevention of health issues and training of these functions. To contribute to this topic, we present in this mini review possible avenues for holistic training approaches for cognitively, physically and mentally fitter and more powerful eSports athletes based on interdisciplinary findings. We discuss exergames as a motivating and promising complementary training approach for eSports athletes, which simultaneously combines physical and cognitive stimulation and challenges in an attractive gaming environment. Furthermore, we propose exergames as innovative full-body eSports-tournament revolution. To conclude, exergames bring new approaches to (physical) eSports, which in turn raise new topics in the growing eSports research and development community.

## Introduction

In today’s digital age, eSports is an up-and-coming, widely discussed and yet mostly recognized new sports genre that is increasingly gaining social, cultural, economic, and scientific interest (e.g., Hallmann and Giel, 2018). eSports (electronic sports) – a form of sport where the primary aspects of the sport are facilitated by electronic systems and the input of players and teams as well as the output of the eSports system are mediated by human-computer interfaces (Hamari and Sjöblom, 2017) – have become a significant part of today’s sports and gaming culture. Teams of players all over the world meet online or, even more spectacular, at big international tournaments to compete against others in a video game battle. According to the International eSports Federation, eSports is officially accepted as a sport in more than 60 countries. Recently, the Olympic Council of Asia approved eSports to be part of the Asian Games in China 2022. Scientists underline this development with scientific rationales for eSports as a showcase of the merit of enhancing cognitive abilities to increase sports performance (Campbell et al., 2018). However, according to the current state of knowledge, much is still unclear if one considers physical and cognitive processes, strains and general health-related issues in eSports athletes as well as specific methods to train, prevent or treat them. With the rise of eSports and the continued emergence of professional eSports leagues, a huge demand exists for new research and development aimed toward understanding and supporting the performance and health-related factors behind this special profession.

To contribute to this uprising topic, we reviewed existing literature in eSports and related fields such as cognitive and movement science as well as interdisciplinary game research. Following, we illustrate how this interdisciplinary knowledge can help to understand and support cognitive, physical and mental processes in eSports athletes and thus serve as a basis for new training approaches towards optimal performance and health in eSports athletes. Finally, we elaborate on and suggest potential avenues, how to compose interdisciplinarily inspired exergame-based training for (physical) eSports athletes.

## eSports Athletes

Several hours per day, professional eSports athletes play the video games they specialized in to improve specific gaming skills including gamepad and keyboard handling, game knowledge as well as strategy and tactics. These long-term cognitive, physical and mental tenses can favor the development of negative health-related side effects.

### Status Quo: eSports Athletes’ Health

On the bodily level, eSports athletes are susceptible to chronic overuse or (sports) injuries such as eye fatigue [excessive exposure to light-emitting diodes (LEDs)] as well as neck, back and wrist pain (Brautigam, 2016; DiFrancisco-Donoghue et al., 2019). These conditions are close to conditions seen in sedentary desk jobs (DiFrancisco-Donoghue et al., 2019). In general, eSports athletes tend to have a higher risk for a diminished health status due to characteristics of eSports (e.g., long-lasting sitting position in front of screens) (Brautigam, 2016; DiFrancisco-Donoghue et al., 2019). On the mental level, eSport athletes might also suffer from diseases such as depression (Rudolf et al., 2016) and burn-out symptoms (Pérez-Rubio et al., 2017). Moreover, psycho-social components can include addictive behavior, personal hygiene issues, social anxiety, and sleep disturbances (DiFrancisco-Donoghue et al., 2019), which in turn can affect the physical, cognitive and mental health state of eSports athletes. Among others, certain cognitive issues are associated with gaming addiction such as cognitive deficits (e.g., impaired executive functioning, hazardous decision-making or deliberative processes) and cognitive biases (e.g., attentional biases, cognitive distortions and dysfunctional cognition) (Billieux et al., 2020). Regarding sleep disturbances, the screen light can impact the natural circadian rhythm and this, in turn, might affect the sleep behavior as well (Tosini et al., 2016). Furthermore, sleep disturbances seem to be related to depression and cognitive impairments, although this relationship varies by age (Alfano et al., 2009). Overall, these health constraints lead to a high drop-out rate and career-ending injuries in professional eSports athletes (DiFrancisco-Donoghue et al., 2019).

### eSports-Specific Performance Requirements

As mentioned before, eSports performance is demanding on several levels and requires different abilities to sustain strenuous tournaments as well as long-lasting, regular daily training sessions. An electronic survey study showed that average eSports athletes train between 3 and 10 h per day (DiFrancisco-Donoghue et al., 2019). Another study surveyed that professional and high-level eSports athletes train 5.28 h every day (Kari et al., 2019). Such a training session can last 3 or more hours in sitting position without standing to take a break (DiFrancisco-Donoghue et al., 2019). Furthermore, eSports performance demands physical effort. Especially during stressful tournaments, the heart rate can extremely increase, e.g., up to 160 to 180 beats per minute (Rudolf et al., 2016). Moreover, supporting musculature of the back and neck are continuously tense in the sitting position and hand muscles are ongoingly needed for fine motor skills. eSports athletes reach up to 400 klicks or keystrokes per minute showing augmented manual dexterity (Green and Bavelier, 2006a; Rudolf et al., 2016). Furthermore, those athletes show highly complex and coordinated skills and movement patterns to interact with their controller devices (e.g., keyboard) (Hilvoorde and Pot, 2016; Campbell et al., 2018).

Even more pronounced in eSports are the cognitive challenges, which is why eSports players are also referred to as “cognitive athletes”. Cognitive resources are needed to learn, train and consolidate specific cognitive abilities, which are necessary to build the cognitive expertise or skill set of eSports athletes. The cognitive workload during eSports game sessions demands various cognitive domains including attention (e.g., dividing and switching attention), perception and information processing (e.g., fast reaction time) and visuo-spatial skills (e.g., navigating in a virtual environment) (Green and Bavelier, 2003, 2006a,b,c, 2007; Bavelier et al., 2012; Bejjanki et al., 2014; Bediou et al., 2018; Chopin et al., 2019; Torner et al., 2019). Furthermore, habitual video game players benefit from enhanced hand-eye coordination (Green and Bavelier, 2006c). To keep in mind, the respective skill set varies depending on the characteristics of the played video game. To properly recall these cognitive skills and to achieve optimal performance, eSports athletes also need a strong foundation of mental and psychological skills (e.g., emotional regulation and attentional control) (Himmelstein et al., 2017).

A clear mind, optimal cognitive abilities and a proper working physical system can be the crucial difference between eSports athletes who win or lose a tournament. Thus, preventive and therapeutic approaches to counteract health-related issues and support long-term health are needed, but also innovative training approaches that specifically train the athletes on the cognitive, physical and mental level. A survey revealed that 55.6% of professional and high-level eSports athletes believe that physical exercise enhances their eSports performance (Kari et al., 2019). Nowadays, professional and high-level eSports athletes perform approximately 1.08 h of physical exercises per day, but rather to increase their healthy lifestyle than to improve their eSports performance (Kari et al., 2019). Nevertheless, a recent study showed that still 40% of the eSports athletes do not participate in any form of physical exercise or have less than 60 min of daily activity (DiFrancisco-Donoghue et al., 2019) indicating that physical exercising is not yet on the daily agenda of every eSports athlete. Regarding cognitive training, besides the daily gaming routine, there is currently no literature discussing potential complementary brain training approaches while some indications exist for mental training in eSports athletes (Nilsson and Lee, 2019). Furthermore, little knowledge exists on the topic of preventive and therapeutic approaches as well as performance-related training (frequency, intensity, time and type of exercises) for eSports athletes (Kari et al., 2019). A current study recommends developing and incorporating a health management model for eSports athletes as well as defining and understanding the medical needs of eSports athletes as it is standard for other athletes (DiFrancisco-Donoghue et al., 2019). As indicated before, one part of such a health management model would include attractive and effective (training) approaches that meet the specific needs of eSports athletes in terms of overall health and gaming performance. To this day, however, no eSports-specific, well-founded and scientifically proven training approaches are known. A promising training approach that could be a beneficial part of a health management model in eSports athletes is combined physical-cognitive training.

### General Beneficial Mechanisms of a Combined Physical-Cognitive Training

Of great interest for eSports athletes are proper cognitive functioning and existing cognitive reserves that could boost gaming performance. Physical exercise can positively affect cognitive functioning by triggering different metabolic brain pathways and mechanism (Thomas et al., 2012; Hötting and Röder, 2013; Voelcker-Rehage and Niemann, 2013; Bamidis et al., 2014; Erickson et al., 2015; Ballesteros et al., 2018; Netz, 2019). Aerobic exercise elevates serum BDNF levels (Vaynman et al., 2004; Knaepen et al., 2010; Lafenêtre et al., 2010; Huang et al., 2014). BDNF is a crucial mediator of the exercise-induced neuroplasticity, and the magnitude of its increase seem to be exercise intensity-dependent (Cotman and Berchtold, 2002; Huang et al., 2014). Further growth factors that might facilitate the effects of aerobic exercise on neuroplasticity are IGF-1 and vascular endothelial growth factor (Trejo et al., 2001; Voss et al., 2013; Maass et al., 2016). In addition, physical exercise has been shown to affect neurotransmitter systems (Lista and Sorrentino, 2010). Moreover, aerobic exercise can increase brain perfusion leading to enhanced supply of oxygen and nutrients (Hötting and Röder, 2013; Maass et al., 2016). Additionally, strength training can create a supportive brain environment via e.g., increased IGF-1 production (Cassilhas et al., 2007; Vega et al., 2010). However, animal studies showed that physical exercise combined with enriched environments (and thus increased cognitive stimulation) might potentiate the positive training effects (Kempermann, 2002; Fabel et al., 2009; Kempermann et al., 2010). Physical exercise facilitates neuroplastic processes while cognitive exercise guides the plastic changes (Fissler et al., 2013; Bamidis et al., 2014). A further crucial factor seems that both components, physical and cognitive, need to be simultaneously present to get the best output (Fissler et al., 2013). A recent review speculated that “[…] incorporating cognitive tasks into motor tasks, rather than separate training of mental and physical functions is the most promising approach to efficiently enhance cognitive reserve […]” (Herold et al., 2018). Next to the cognitive improvement by a combined physical-cognitive approach, exercising can also trigger general physical effects depending on the physical components that are integrated into the training [e.g., aerobic components can enhance the cardiovascular system (Cornelissen and Fagard, 2005), strength components can improve the musculoskeletal system (Kristensen and Franklyn-Miller, 2012; Ciolac and Rodrigues-da-Silva, 2016), and motor components can influence coordination and balance skills (Pless and Carlsson, 2000; Kümmel et al., 2016)]. For eSports athletes, these potential adaptations can positively influence their general health status and that, in turn, can support their gaming performance in training and tournament situations.

An innovative training approach that could take up the holistic body’n’brain training in the field of eSports athletes and that could be a combined physical-cognitive and motivating addition to the conventional training approaches are exergames. Due to their playful training approach and the typical setup, exergames could offer a somewhat familiar and thus particularly attractive training approach for eSports athletes while potentially achieving effective cognitive, physical and mental outcomes for eSports athletes.

## Perspectives on Exergames in eSports

Exergames are single or multiplayer games that are controlled by physically active body movements (Oh and Yang, 2010; Mueller et al., 2016). Some require more physical effort than others (full-body movements versus moving single body parts). Exergames, which are also known as movement-based games (Isbister and Mueller, 2015), active video games (Biddiss and Irwin, 2010) or exertion games (Mueller et al., 2016), can be played in different settings. Typically, a player who physically interacts with a motion-based controller technology moves in front of a screen, which displays a virtual game scenario. Thus, commercially available exergame platforms such as the Nintendo Wii, the Sony Move or the Microsoft Kinect and their corresponding games have successfully been turning living rooms into playful training settings for about 10 years now (Mueller et al., 2016; Martin-Niedecken and Mekler, 2018). Besides virtual exergame environments, we also find exergame scenarios, which, similar to classic sports games, can be played analogously in the physical space with optional technical aids (e.g., physical obstacles or devices) and thus completely do without the classic player-screen setting (e.g., Segura et al., 2013). Apart from the entertainment market, game-based training and therapy applications further establish themselves in the fitness and rehabilitation industry (e.g., Martin-Niedecken et al., 2019).

### Evidence in Exergame Training

So far, studies in the field of exergaming have investigated various effects of commercially available and specifically developed exergames in different target population such as children, adolescents, seniors or patients. Results deliver indications for effects on the cognitive (e.g., executive functions, attention and visual-spatial skills) (Staiano and Calvert, 2011; Best, 2015; Benzing et al., 2016; Mura et al., 2017; Stojan and Voelcker-Rehage, 2019; Xiong et al., 2019), physical (e.g., energy expenditure, heart rate, and physical activity) (Staiano and Calvert, 2011; Sween et al., 2014; Best, 2015; Kari, 2017) and mental (e.g., social interaction, self-esteem, motivation, and mood) (Staiano and Calvert, 2011; Li et al., 2016; Joronen et al., 2017; Lee et al., 2017; Byrne and Kim, 2019) level. Generally, exergames are very well known for their playful combination of physically and cognitively challenging tasks and thus provide dual domain training, which indicates to have greater effects compared to traditional training approaches (Schättin et al., 2016; Ballesteros et al., 2018; Egger et al., 2019; Stojan and Voelcker-Rehage, 2019). Nevertheless, more studies are needed that examine the effects of long-term training periods.

Besides the cognitive, physical and mental effectiveness of exergame training, it is further known for its appealing and motivating impact, especially in physically less active populations (e.g., Lu et al., 2013; Kappen et al., 2019). By offering different players [with different motivational types (Tondello et al., 2019)] an audio-visual and narrative appealing, immersive game scenario, exergames enable a shift of the (cognitive) focus of the player to the playful experience, making it easy to engage in a physically challenging training (Martin-Niedecken et al., 2019). Exergames have successfully been shown to increase training adherence (e.g., Valenzuela et al., 2018), long-term motivation (e.g., Macvean and Robertson, 2013), engagement (e.g., Lyons, 2015), immersion (e.g., Lu et al., 2013), and flow experience (e.g., Martin-Niedecken and Götz, 2017) in players from different populations. However, further research and development work is needed to fully explore and understand the effects of various exergame design elements on players’ gameplay and training experience considering the physical, cognitive and mental level.

### Exergames – Innovative Training Tools in eSports Athletes

**FIGURE 1:**
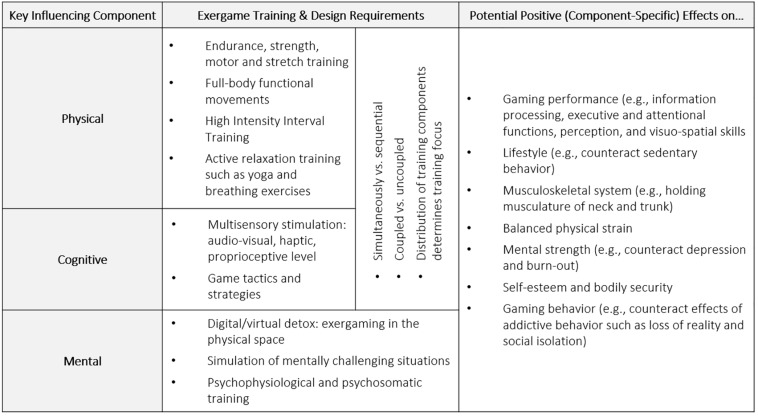
Summary of avenues for the development of eSports-specific exergame concepts and the effects of selected physical, cognitive and mental training as well as design components on eSports athletes’ performance and health.

If designed properly in terms of effectiveness and attractiveness, exergames allow for innovative, motivating and holistic training approaches, which may be extremely suitable and beneficial in eSports athletes to keep and maintain their cognitive, physical and mental processes and thus to increase their eSports-related performance and health. Multiple interdisciplinary design guidelines provide well-funded recommendations and considerations for the design of effective (Wüest et al., 2014; Hardy et al., 2015; Hoffmann et al., 2016; Benzing and Schmidt, 2018) and attractive (Sweetser and Wyeth, 2005; Sinclair et al., 2007, 2009; Segura et al., 2013; Mueller and Isbister, 2014; Kajastila and Hämäläinen, 2015; Isbister, 2016; Mueller et al., 2016) exergames, which immerse the player in a motivating and flowing workout experience in front of a screen or in a physical play space. In the context of eSports, however, exergames are not yet used or evaluated as potential training tools. In order to meet the previously described diverse physical, cognitive and mental needs of eSports athletes, exergames must meet certain requirements that are aimed at the specific requirements and general health aspects, which in turn have a positive influence on eSports performance. Following exemplarily avenues for potential eSports-specific exergame concepts including physical, cognitive and mental components are described (Figure 1).

Generally, exergames allow for the development of target audience-specific holistic body’n’brain training concepts featuring full-body movements which follow specific design and training principles. To counteract health-related symptoms of eSports athletes’ mainly sedentary activities and one-sided physical strain, exergames could feature full-body functional movements (Weiss et al., 2010) with a strong focus on strength training (e.g., holding musculature, trunk musculature, and strength endurance) and flexibility elements (e.g., coordination and stretching). To support performance-related physical-cognitive processes particularly during tournament situations, exergames should further feature cardio-vascular training elements (e.g., high intensity interval training) (Farrow et al., 2019). On the cognitive level, exergames provide several options to train executive functions (e.g., inhibition and flexibility) and attentional functions (e.g., divided and selective attention) via various multi-sensory stimuli on the audio-visual (Ekman, 2008), haptic (Shaw et al., 2017) or proprioceptive level (Moran et al., 2016). Exergames further allow for real-time adaptations of both the cognitive and the physical challenge, based on each player’s personal fitness, gaming skills and performance and thus can provide an individualized, optimally balanced training mode (Sinclair et al., 2007).

Furthermore, due to the combination of gaming and exercising, exergames can positively affect eSports athletes’ mental state, especially if they suffer from depression and thus feel completely unmotivated to participate in any physical activity (Li et al., 2016). The implementation of specific game mechanics, feedback loops and design parameters may further be suitable to train certain game tactics/strategies, which are needed in eSports games and to extend the very specific skill profile of eSports athletes. Among others, exergames allow simulating socially and bodily competitive multiplayer situations. This might help eSports athletes to mentally handle stressful situations (Argelaguet Sanz et al., 2015) and train mental strength (Kamel Boulos, 2012) by triggering bodily security and self-esteem (Gerling et al., 2014), which are valuable attributes when it comes to eSports tournaments. The implementation of more calming and active relaxation training concepts such as yoga or breathing exercises could further help to target anxiety, stress and other psychophysiological, psychosomatic and mental issues (Van Rooij et al., 2016; Patibanda et al., 2017). Moreover, eSports athletes could benefit from more analogous exergame designs, as they take the players out of the often very engaging virtual world and focus on the real environment, while still maintaining the playful character and thus particularly counteract potential negative effects of gaming addiction.

Finally, exergames can also serve as assessment tools. Next to various physical and game performance-related input parameters, exergames could track training effects on specific skill levels or allow for planning and analyzing individual training sessions. Thus, the body-centered characteristics of exergames may also be a supportive tool for eSports coaches. However, not every commercially available exergame can be used as an additional eSports training tool. To provide a benefit for eSports athletes’ gaming performance and health, specifically modified or newly developed, effective and attractive exergames are needed, which are co-designed and evaluated by an interdisciplinary team of experts from the fields of eSports, game design and research, movement and cognitive science as well as psychology.

### eSports Becomes Physical – The Exergame-Based Future of eSports?!

**FIGURE 2:**
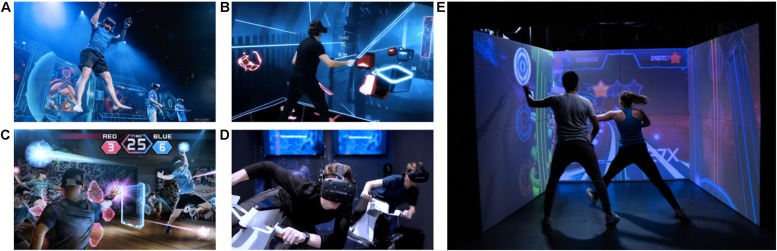
Augmented, virtual and mixed reality technologies make eSports more and more physical. **(A)** Echo Arena, **(B)** Beat Saber, **(C)** Hado AR, **(D)** Icarace, and **(E)** the ExerCube are examples of exergames, which require not only fine motor and cognitive skills, but also certain levels of physical effort and which are or will be performed in eSports tournaments. Hence, new physical-cognitive training approaches are not only needed to sustain and improve eSports athletes’ gaming performance and general health, but also to develop physical eSports concepts.

Besides their potential application as an additional training tool in eSports athletes, exergames further open up new possibilities for physical eSports leagues (Kajastila and Hämäläinen, 2015). To be suitable as a competitive discipline, exergames again must fulfill certain requirements such as standardized setups and scenarios or multiplayer balancing (e.g., Bayrak et al., 2017). Today, immersive Virtual and Augmented Reality technologies allow players to physically engage in games. eSports is following this trend and thus more and more physical game genres are establishing themselves in professional leagues, which are played against each other in the context of larger tournaments (Figures 2A–E).

Given the current trend, it is even more important to provide athletes with specific exergame training tools besides the games they specialized into. One exergame example which unites various of the previously mentioned requirements, such as an effective full-body functional training, is scalable from moderate to high intensity, provides an attractive and physically immersive gaming environment, and was designed and evaluated by an interdisciplinary team of sports scientists and game designers, is the ExerCube (Martin-Niedecken and Mekler, 2018; Martin-Niedecken et al., 2019) by Sphery Ltd. (Figure 2E).

## Conclusion

Today eSports is a widely discussed and omnipresent phenomenon. eSports athletes compete in major tournaments in front of an audience of millions. To be in top form and able to cope with this situation as well as to counteract general health issues caused by the very special athlete profiles, eSports athletes need optimal cognitive, physical and mental abilities. However, a holistic health management system for eSports athletes is missing. Findings from interdisciplinary work in related fields such as cognitive and movement science or game research will provide potential avenues for the development of holistic body’n’brain training approaches for eSports athletes. A very promising and innovative training approach is exergaming, which combines physical and cognitive training in an attractive gaming environment. Considering the game design and training principles as well as eSports specific requirements, exergames could serve as an additional training option to holistically support gaming performance and general health in eSports athletes. However, further interdisciplinary research and development work is needed to understand and meet the various requirements of eSports athletes and to unite them into exergames, which then may serve as a training tool and an eSports genre. To conclude, exergames bring new approaches to (physical) eSports, which in turn raise new topics in the growing eSports research and development community.

## Author Contributions

AM-N and AS equally contributed to the conceptualization of the mini review, compilation and review of relevant related work, and writing of the manuscript.

## Conflict of Interest

AM-N is the co-founder and CEO of the startup company Sphery Ltd. who developed the ExerCube. No revenue was paid (or promised to be paid) directly to AM-N or the research institutions. The remaining author declares that the research was conducted in the absence of any commercial or financial relationships that could be construed as a potential conflict of interest.

## Abbreviations

BDNF: brain-derived neurotrophic factor
IGF-1: insulin-like growth factor 1.
